# Transapical Deployment of Thoracic Stent Graft for Ascending Aorta Coronary Bypass Pseudoaneurysm in a Patient with Prosthetic Aortic Valve

**DOI:** 10.1055/s-0039-1687864

**Published:** 2019-07-22

**Authors:** Michele Antonello, Augusto D'Onofrio, Marco Zavatta, Giambattista Isabella, Carlo Maturi, Michele Piazza, Gino Gerosa

**Affiliations:** 1 Department of Cardiac, Thoracic and Vascular Sciences, Vascular and Endovascular Surgery Clinic, School of Medicine, Padova University, Padova, Italy; 2 Division of Cardiac Surgery, Department of Cardiac, Thoracic and Vascular Sciences, School of Medicine, Padova University, Padova, Italy; 3 Division of Cardiology, Department of Cardiac, Thoracic and Vascular Sciences, School of Medicine, Padova University, Padova, Italy

**Keywords:** transapical, ascending aorta, CABG pseudoaneurysm

## Abstract

The authors describe the transapical deployment of a thoracic endograft to exclude a saphenous vein graft proximal anastomotic pseudoaneurysm following coronary artery bypass grafting (CABG) in a 63-year-old male with a prosthetic aortic valve. A standard thoracic endograft has been deployed via transapical access after percutaneous transluminal coronary angioplasty of the native vessel perfused by the patent CABG. The procedure was uneventful; an 8-month computed tomography scan showed complete exclusion of the pseudoaneurysm with patency of supra-aortic trunks.

## Introduction

Ascending aorta and aortic arch aneurysms still represent one of the most complex conditions to be managed.

Open surgery is the gold standard in high risk patients; although surgical techniques have improved considerably over time, the need for cardiac arrest and extracorporeal circulation carries significant peri- and postoperative morbidity and mortality rates.

On the other hand, thoracic endovascular aortic repair (TEVAR) has evolved rapidly as the standard treatment for lesions of the descending aorta, because of its high technical success rate and low operative risk. Therefore, hybrid techniques with surgical debranching of supra-aortic vessels followed by TEVAR are today a valid alternative to open repair. 1

Besides that, providing patients an endovascular treatment option for ascending aorta is still complex and represent one of the last and biggest challenges of the endovascular era. New trials are testing the role of endoprosthesis dedicated to ascending aorta and aortic arch even if not yet available commercially. 2 At this time, endovascular solutions are applied only to compassionate cases in need of urgent repair; therapeutic options are based on alternative adaptation of available devices in a case-by-case fashion.

## Technique and Results

**Fig. 1:**
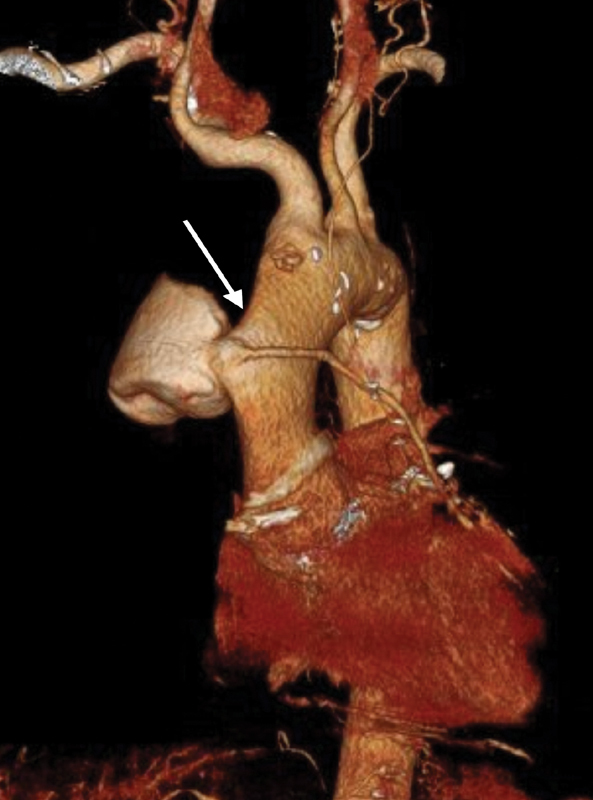
Preoperative computed tomography (CT) angiogram three-dimensional (3D) reconstruction; arrow indicates the anastomotic pseudoaneurysm and the patent venous graft.

The technique is demonstrated in a 63-year-old male, with dilatative cardiomyopathy and a 32% ejection fraction, who underwent open chest surgery with prosthetic aortic valve replacement (Magna Ease 23 mm, Edwards Lifesciences, Irvine, CA), mitral valve repair (St. Jude Rigid Saddle 32 mm, St. Jude Medical, St. Paul, MN), and a double coronary artery bypass grafting (CABG) (left internal mammary artery [LIMA] to D2 and great saphenous vein to D1) due to severe stenosis of the proximal tract of the left anterior descending (LAD) coronary artery involving the origin of D1. LAD was intraoperatively found to be intramyocardial and the operator decided to revascularize D2. A 6-month follow-up, transthoracic echocardiogram demonstrated the presence of an ascending aorta dilatation, subsequently confirmed by a computed tomography (CT) angiogram ( Fig. 1 ). In particular, a 73 × 70 mm saccular pseudoaneurysm of the ascending aorta at the proximal anastomosis of the venous graft to D1 was detected.

Because of the high surgical risk related to clinical and anatomical reasons, the patient was deemed unsuitable for a redo open surgery and an endovascular solution had to be considered. The site of the aortic lesion had a suitable landing zone above the sinotubular junction of 25 mm in length and a landing zone before innominate artery origin of 20 mm in length, with a homogeneous aortic diameter of 37 mm and a total length to be covered of 100 mm. A single tube Gore Conformable Thoracic Aortic Graft (TGE 45–45–10; WL Gore and Assoc. Inc., Flagstaff, AZ) was planned with a 20% oversizing. Owing to the presence of an aortic prosthetic valve, a femoral access with the shaft tip crossing valve leaflets in a retrograde fashion was to be avoided, and a transapical approach through a mini left thoracotomy was considered. Moreover, one of the major concerns was that the endovascular exclusion of the pseudoaneurysm would also cause CABG occlusion with high risk for acute myocardial infarction. After multidisciplinary discussion with interventional cardiologist, we decided for a preventive attempt of percutaneous transluminal coronary angioplasty (PTCA) of the native LAD at its origin. The patient accepted the therapeutic program proposed. He first underwent successful PTCA of the origin of the native LAD; after 48 hours, endovascular exclusion of the aortic pseudoaneurysm was attempted.

**Fig. 2:**
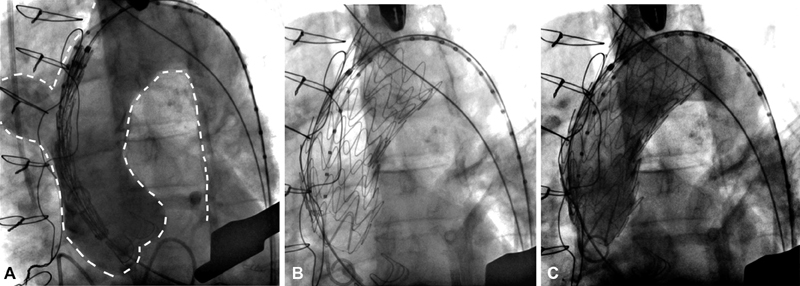
( **A** ) Angiogram showing the ascending aorta pseudoaneurysm and the positioning of a guidewire from a right omeral access to identify the exact origin of the innominate artery from the arch. ( **B** ) Correct positioning of the endograft into the ascending aorta. ( **C** ) Intraoperative final angiogram showing the exclusion of the ascending aorta pseudoaneurysm.

The technique was performed in a surgical hybrid suite, under general anesthesia, in presence of vascular and cardiac surgeons. Through a left anterolateral mini-thoracotomy along the fifth intercostal space, exposure of the left ventricular apex was obtained in a standard fashion. The pericardial sac was opened, and a double purse-string suture, reinforced with Teflon pledgets, was performed before apex puncture. We decided to choose as puncture site an area slightly lateral and above the anatomical apex, where the myocardial tissue seemed thicker. After preparation of the sutures, the patient was heparinized with an activated clotting time goal of 300″. The left ventricle was then punctured with a 19-G needle and access to the aorta obtained through a 24 F Dryseal introducer (WL Gore and Assoc. Inc.) advanced over the prosthetic aortic valve, to avoid any friction with the valve leaflets during maneuvers of introduction, positioning, and removal of endograft devices. From a transfemoral venous access, a pacing wire has been advanced into the right ventricle. As the proximal landing zone of the endograft has been planned to be just proximally the innominate artery, a guidewire has been inserted through an omeral access to identify its origin and eventually protect its patency ( Fig. 2A ).

**Fig. 3:**
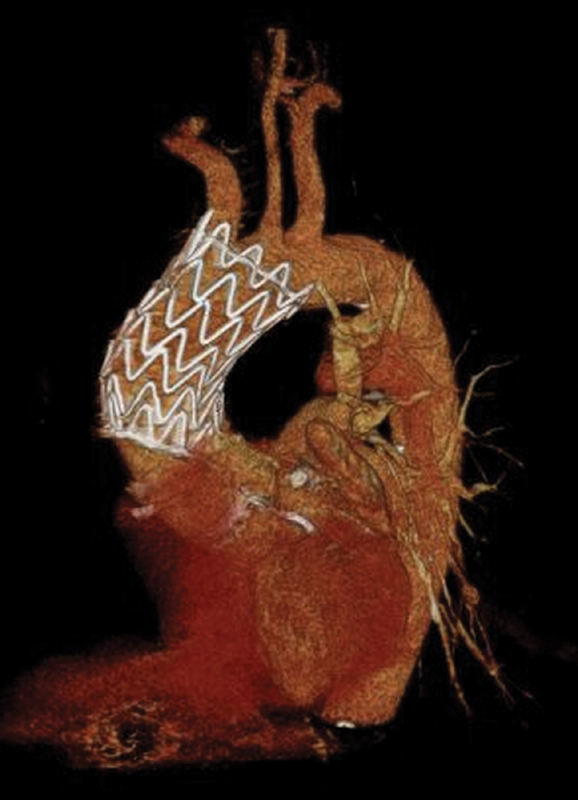
Computed tomography angiogram three-dimensional (3D) reconstruction at 8 months after endovascular repair.

We advanced a guidewire into the ascending aorta under fluoroscopic guidance. Hereafter, a Lunderquist extra-stiff guidewire (Cook Medical Inc., Bloomington, IN) was placed and the endoprosthesis, a Gore C-TAG TGE45–45–10 (W. L. Gore and Associates, Flagstaff, AZ), advanced in the ascending aorta. To guarantee a precise deployment and minimize windsock effect, we deployed the endograft under rapid pacing stimulation and ventilation with temporary arrest. At final intraoperatory angiography, the endograft appeared well positioned, with complete exclusion of the pseudoaneurysm and patency of the supra-aortic trunks ( Fig. 2B and C ). Hemostasis was achieved by tightening the purse-string sutures while removing sheats from the left ventricle. A short period of rapid pacing stimulation during suture tightening facilitated hemostasis, lowering systolic blood pressure. Finally, pericardium was closed over the apex and a left lateral chest tube was placed before wound closure in a standard fashion. The procedure was uneventful, and the overall procedure time was 90 minutes. Postoperative period was free from complications, intensive care unit stay was 1 day; a CT angiogram at 8 months demonstrated the complete exclusion of the pseudoaneurysm ( Fig. 3 ).

## Discussion

Saphenous vein graft pseudoaneurysm is a rare complication of CABG surgery and may lead to life-threatening scenarios. The reported incidence in the literature is less than 1%. 3 Because of the rarity of the disease there is lack of randomized trials and of standardized treatment procedures.

These are often patients with several comorbidities and their exposure to high surgical risks related to redo sternotomy, cardiac arrest with cardiopulmonary bypass is a major issue.

An endovascular option, with exclusion of the ascending aorta CABG pseudoaneurysm, may be a simple and valid alternative; however, several challenges have to be considered.

First of all, the available thoracic endograft in the market is designed for descending aorta and has significant limitations when considered for use in a different site such as the ascending aorta. In particular, in case of tall patients or extremely tortuous aortic anatomies, the delivery system may be too short to allow deployment in the ascending aorta from a standard femoral access. Another crucial point is that often the endograft length is not adequate to fit with the ascending aorta. The length of the ascending aorta from the sinotubular junction to the innominate artery varies from 50 to 100 mm, with the majority of cases with a length between 60 and 70 mm. On the other side, all the commercially available TEVAR devices have a minimum covered length of 100 mm, or 77 mm considering also the proximal extension component.

Finally, the final tip of standard thoracic endograft is rigid and long, and not adequate to cross multiple times the aortic valve, increasing the risk for severe traumatic lesions of the ventricle and valve malfunctions.

To overcome all these limitations, some authors 2 already reported their preliminary experience in a small cohort (10 patients) with a new dedicated ascending aorta device (Zenith AscendEndograft; William Cook Europe, Bjaeverrskov, Denmark); unfortunately, this is still an investigational device.

Therefore, today the endovascular treatment of these rare cases is based on alternative adaptation of off-the-shelf devices in a case-by-case fashion.

Different alternative access options for thoracic endograft have been reported by some authors. Rayan et al 4 used a left axillary access, but in this case was to be avoided because of the risk of temporary LIMA bypass occlusion.

Access from the cardiac apex through a mini thoracotomy is routinely performed in any cardiac surgery center, where it is widely used for transcatheter aortic valve implantation in patients with poor femoral access, and is recognized as a safe and standardized access. 5

To our knowledge, since the first description of a transapical access for the deployment of a thoracic endograft in 2008 by MacDonald et al, 6 only few TEVAR are described with this access, 7 and only one is reported for the treatment of an anastomotic CABG pseudoaneurysm of the ascending aorta. 8 However, it is not described yet in the literature a transapical deployment of an endograft in patient with prosthetic aortic valve.

## Conclusion

In conclusion, the use of a transapical access for the deployment of an ascending thoracic aorta endograft seems safe and feasible also in case of prosthetic aortic valve; it may be used to extend indications for aortic repair in selected patients who are at high surgical risk and cannot wait for a custom endograft. A multidisciplinary approach represents the key for adequate case planning and clinical success in these rare and unusual pathologies.
